# High temperature requirement A3 (HTRA3) expression predicts postoperative recurrence and survival in patients with non-small-cell lung cancer

**DOI:** 10.18632/oncotarget.9173

**Published:** 2016-05-04

**Authors:** Jingya Zhao, Jing Zhang, Xin Zhang, Mingxiang Feng, Jieming Qu

**Affiliations:** ^1^ Department of Pulmonary Medicine, Huadong Hospital, Fudan University, Shanghai, China; ^2^ Department of Pulmonary Medicine, Zhongshan Hospital, Fudan University, Shanghai, China; ^3^ Department of Thoracic Surgery, Zhongshan Hospital, Fudan University, Shanghai, China; ^4^ Department of Pulmonary Medicine, Ruijin Hospital, School of Medicine, Shanghai Jiaotong University, Shanghai, China

**Keywords:** HTRA3, postoperative recurrence, non-small-cell lung cancer, invasion, immunohistochemistry

## Abstract

Non-small-cell lung cancer (NSCLC) is the leading cause of cancer-related death worldwide, and its recurrence rate after complete resection is high, owing to local or distant metastases. Low expression of high temperature requirement A3 (HTRA3) has been reported to promote tumorigenesis, diminish the effects of anti-tumor treatments, and correlate with a malignant phenotype. To assess the involvement of HTRA3 in the prognosis of postoperative NSCLC, we obtained tumors from 78 patients who had undergone complete surgical resection, and immunohistochemically examined them for HTRA3 expression. HTRA3 was significantly down-regulated in lung cancer tissues compared with normal lung tissues, and only six tumor cases(7.7%) exhibited relatively high levels of HTRA3 (*P* < 0.001). Notably, high-HTRA3 patients were at significantly lower risk of postoperative recurrence than low-HTRA3 or HTRA3-negative patients (0% *versus* 31.2% and 35.0%; *P* = 0.044, 0.029, respectively). High expression of HTRA3 also independently indicated longer disease-free survival in Cox regression analysis (hazard ratio 0.39, 95%CI 0.16-0.95, *P* = 0.038). Ectopic expression of the long isoform of HTRA3 attenuated the invasion of an NSCLC cell line in a Transwell assay, while knockdown of *HTRA3* had the converse effect. Thus, HTRA3 suppresses tumor cell invasiveness and may serve as a prognostic biomarker for postoperative recurrence or survival in NSCLC.

## INTRODUCTION

Lung cancer continues to be the most frequently occurring type of cancer and one of the most common causes of cancer death worldwide [1]. Non-small-cell lung cancer (NSCLC) accounts for approximately 80% of cases. Although surgical resection with a curative intent is considered to be the standard of care for early-stage NSCLC, recurrence after resection has been reported in 30-75% of all cases from stage I to stage III [2–5]. The poor postoperative prognosis of NSCLC is mostly due to the development of local and distant metastases. However, for patients undergoing surgical treatment, there are few definite prognostic markers, aside from anatomic or pathological factors [6]. Thus, the identification of reliable biomarkers that can be used to better stratify individuals at increased risk of relapse after complete resection of early-stage NSCLC is important to improve clinical outcomes.

High temperature requirement A3 (HTRA3) belongs to the well-conserved HTRA family of oligomeric serine proteases [7]. Members of the HTRA family have different biological functions; for instance, the *Escherichia coli* HTRA (Degp) acts as a protease that degrades misfolded proteins at high temperatures and as a molecular chaperone at low temperatures [8]. In humans, four HTRAs (HTRA1-4) have been identified [7]. Two variants of human *HTRA3* mRNA (long and short) have been identified, corresponding to two HTRA3 protein isoforms (49kDa and 39kDa, respectively) produced through alternative splicing. The long isoform of HTRA3 (HTRA3-L) has four distinct domains, the insulin-like growth factor binding (IGFB) domain, Kazal-type protease inhibitor domain, trypsin-like serine protease and postsynaptic density protein 95-Discs large-Zona occuldens 1 (PDZ) domain. The 39-kDa short isoform (HTRA3-S) lacks the PDZ domain, and in its place has a unique sequence of seven amino acids at the C-terminus, which is encoded by a separate exon [9]. Of the four human HTRA family members, HTRA3 shares its domain organization with HTRA1. Previous studies have suggested that HTRA1 is a tumor suppressor: it is down-regulated in various cancers, and its down-regulation is associated with tumor proliferation, chemotherapy resistance and a metastatic phenotype [10–12]. The expression of HTRA3 is also dramatically reduced in endometrial and ovarian cancers [13–15]. In one lung cancer study, HTRA3 expression promoted mitochondrial cell death and chemotherapy-induced cytotoxicity [16]. However, the potential involvement of HTRA3 in the prognosis of surgically resected early-stage NSCLC has not been fully explored.

In the present study, we analyzed the expression of HTRA3 in NSCLC and assessed whether HTRA3 expression correlated with NSCLC recurrence or prognosis in postoperative patients. Moreover, by using NSCLC cell lines, we further investigated whether HTRA3 influenced the progression of NSCLC by promoting or inhibiting tumor cell invasion.

## MATERIALS AND METHODS

### Patients and study design

Between 2007 and 2008, tumor specimens from 297 consecutive patient operations at Shanghai Zhongshan hospital were submitted for our study. Four patients who received preoperative radiotherapy were removed. Of the remaining 293 patients, nine with positive surgical margins and 206 with postoperative adjuvant therapy before tumor relapse were also excluded. Thus, 78 NSCLC tissues from complete tumor resections were suitable for analysis. Clinical information was derived from the electronic medical record database, and postsurgical tumor staging of the patients was performed based on the international staging system. Control lung tissues (*n* = 12) were obtained from patients after surgeries for non-cancerous pulmonary diseases such as tuberculosis (*n* = 4), pneumonia (*n* = 7) and bronchiectasis (*n* = 1). These patients consisted of 8 (66.7%) men and 4 (33.3%) women, with the age range from 47 to 76 years old. The ethical committee of Shanghai Zhongshan Hospital approved the current research, and each patient provided informed consent.

Postoperative follow-ups were scheduled at one month, two months and every three months thereafter during the first two years after surgery, and then every six months thereafter, or more frequently if needed. Follow-up studies included a physical examination, carcinoembryonic antigen analysis, computed tomography, ultrasound examination and magnetic resonance imaging, as well as fiberoptic bronchoscopy if necessary. Tumor relapse was established based on clinical, radiological or histological diagnosis, and the sites and times of the tumor relapses were recorded. The follow-up time was calculated until December 2011. Disease-free survival (DFS) was measured from the date of surgery until tumor relapse or death. Overall survival (OS) was measured from the date of lung cancer surgery until the time of death.

### Immunohistochemistry for HTRA3 expression

Four-micrometer sections were cut from the surgically resected, paraffin-embedded tissue samples. The slides were baked at 55°C overnight, then deparaffinized in xylene and rehydrated through treatment with a graded series of ethanol concentrations. Endogenous peroxidase activity was blocked via incubation of the slides in 3% H_2_O_2_ in 1× high-salt Tris-buffered saline for 15 min at room temperature. Nonspecific staining was blocked by incubation with10% fetal calf serum for 20 min. The polyclonal rabbit antibody against HTRA3 was purchased from Abcam (Cambridge, UK), and the slides were incubated with it for 1 h at room temperature. After serial washing steps and anti-rabbit secondary antibody incubation, diaminobenzidine was used as the final chromogen and hematoxylin was used as the nuclear counterstain. Rabbit IgG (at the same concentration as the antigen-specific antibody) was used as a negative control. Considering the experimental variability and the stability of the antibody, we performed the immunohistochemistry twice for each case.

Immunostaining was scored semi-quantitatively by two separate observers. An intensity score was assigned from 0 (no staining) to 3 (maximal staining intensity). The percentage of positive tumor cells was also scored: no positive cells as 0, 1-10% of positively stained cells as 1, 11-50% as 2, 51-80% as 3, and 81-100% as 4. The intensity score was multiplied by the percentage score in each case to yield a total semi-quantitative result between 0 and 12 [17].

### Cell lines and reagents

Human lung adenocarcinoma A549 and SPC-A1 cell lines were purchased from the American Type Culture Collection (ATCC, Manassas, VA, US). A549 was cultured in Dulbecco's Modified Eagle's Medium (DMEM) supplemented with 10% fetal bovine serum (FBS; GIBCO, Grand Island, NY, US). SPC-A1 was cultured in high-glucose RPMI medium 1640 containing the same supplements. All cell lines were maintained at 37°C in the presence of 5% CO_2_. The antibody against actin was purchased from AbMART (Shanghai, China).

### Stable transfection and lentiviral transduction

Human full-length *HTRA3-L* cDNA was purchased from Obio Technology (Shanghai, China) and subcloned into the lentiviral expression plasmid pLenti-CMV-EGFP-PGK-Puro. The *HTRA3-L* expression vector or the empty vector was transfected into HEK 293T cells according to the manufacturer's instructions in order to produce the recombinant lentivirus. Then, the recombinant lentivirus containing *HTRA3-L* or empty virus was added to the A549 and SPC-A1 cells with 5.0ug/mL polybrene. After 48h, the cells were resuspended in fresh puromycin-containing medium (1.0ug/mL puromycin) and selected for 72h. Surviving cells were subjected to Western blotting so that HTRA3-L expression could be assessed. Thus, we generated A549 and SPC-A1 cell lines stably overexpressing *HTRA3-L*, as well as the corresponding vector control cell lines.

### RNA interference

Chemical small interfering RNAs (siRNAs) were synthesized by GenePharma, Ltd (Shanghai, China). A549 cells were plated in six-well plates and allowed to grow overnight so that they reached approximately 50% confluence. Then, the cells were transfected with *HTRA3*-specific siRNAs, *HTRA3-L*-specific siRNAs or a scrambled control siRNA by means of the Lipofectamine RNAiMAX reagent (Invitrogen) according to the manufacturer's instructions. Thirty-six hours later, some of the transfected cells were subjected to the Transwell assay, and the remaining cells were collected 12h later so that the efficacy of RNA interference could be determined by Western blotting.

### Western blot analysis

Cells were harvested and rinsed with cold phosphate-buffered saline, and the total protein was obtained with a lysis buffer as detailed previously [18]. The protein concentration was determined by the BCA method, and equal amounts of protein were electrophoresed in a sodium dodecyl sulfate-polyacrylamide gel and transferred onto a nitrocellulose membrane. The membrane was blocked with 3% bovine serum albumin in Tris-buffered saline-Tween 20 for 1.5h at room temperature and incubated with the appropriate primary antibody at 4°C overnight. The membrane was washed and incubated with a horseradish peroxidase-conjugated secondary antibody (1:2000 dilution) for 1.5h at room temperature. The immunoreactive protein bands were visualized with enhanced chemiluminescence. β-actin was used as the protein loading control and all experiments were performed at least three independent times.

### Transwell assay

The Matrigel invasion assay was performed in a 24-well Transwell chamber with 8-um pore-size Transwell filters (Corning, 6.5-mm diameter). The cell-culture inserts were coated with 0.1mL of Matrigel (1:19 dilution; Sigma, Louis, MO, US). Then, A549 or SPC-A1 cells were trypsinized, and single-cell suspensions were placed into the upper Transwell chamber (1×10^5^ cells per well) in 0.1mL of serum-free medium. Medium supplemented with 10% FBS was added to the lower chamber as the chemoattractant. After 15h of incubation in 5% CO_2_ at 37°C, cells from the upper surface of the chamber were removed and the invasive cells on the lower surface of the filter were fixed, stained and counted in at least six random fields under a microscope. The experiments were performed in duplicate.

### Statistical analysis

All statistical analyses were carried out with SPSS software version 16.0(SPSS Inc., Chicago, IL). Differences in major clinicopathologic features and prognostic factors among the categorized groups with varied HTRA3 expression were calculated with either the χ2 test or Fisher's exact test. The Kaplan-Meier method was used to calculate the DFS and OS, and a log-rank test was used to determine the statistical significance of the differences in survival among the patient subgroups. Multivariate Cox regression analysis was used to identify significant independent prognostic factors. Data from the Transwell assay were represented as mean ± standard deviation (SD), and differences between groups were assessed with Student's t-test. Significance was established when P values were less than 0.05. All tests were two-sided.

## RESULTS

### Expression of HTRA3 in normal lung and NSCLC tissues

**Figure 1:**
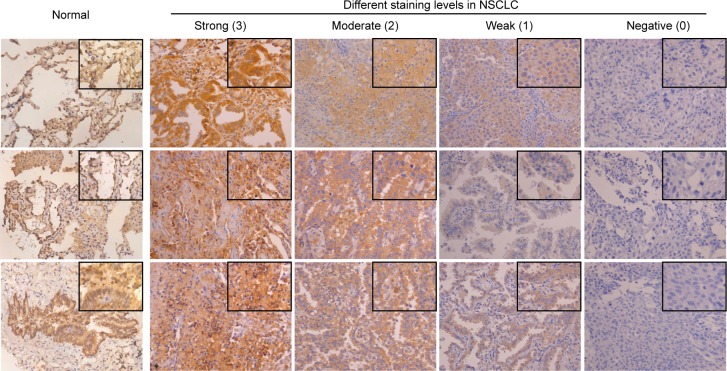
Immunohistochemical staining of HTRA3 protein in normal and lung cancer tissues Representative staining patterns for the expression of HTRA3 protein in normal and cancerous tissues are shown. All the normal cases displayed evident immunoreactivity. The staining intensity in NSCLC was scored from 0-3. (×200 magnification; areas in squares are ×400 magnification).

**Figure 2:**
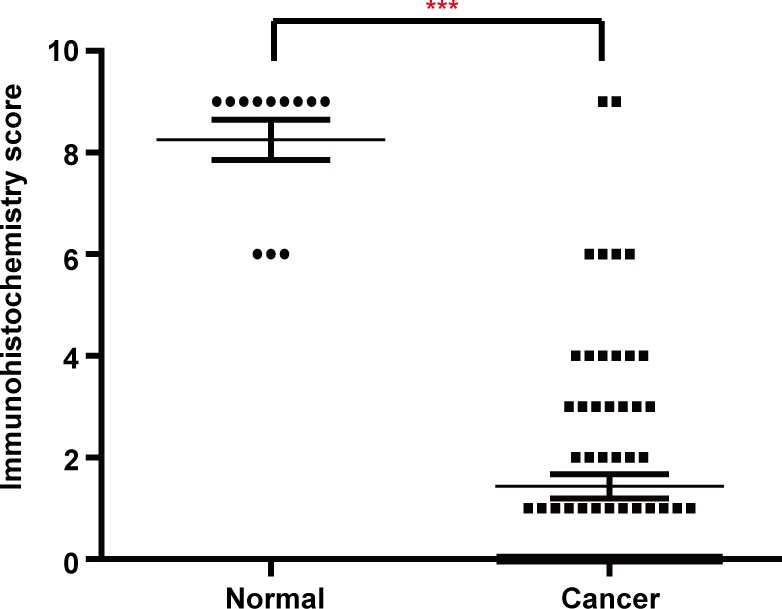
Scatter plot of the immunostaining scores for HTRA3 in normal and lung cancer tissues Semi-quantitative analysis indicated that HTRA3 levelsweresignificantly lower in lung cancer tissues than in normal tissues. ***P < 0.001.

The immunohistochemical analysis revealed cytoplasmic immunoreactivity in all the normal samples. A total of 78 NSCLC patients were enrolled in this study, and their clinical characteristics are shown in Table 1. Among the tumor specimens from these patients, 38 (48.7%) expressed HTRA3 protein. Representative immunoreactivity intensities of normal and tumorous specimens are shown in Figure 1 (negative staining control for each tumorous specimen is in Supplementary Figure 1). In our semi-quantitative analysis, the expression score for HTRA3 protein was significantly lower in lung cancer specimens than in non-NSCLC specimens (*P* < 0.001, Figure 2).

**Table 1:** Patients characteristics (n = 78)

Characteristics	*n*	(%)
**Age (yr)**		
**Median**	61.5	
**Range**	27-78	
**Sex**		
**Male**	53	(67.9)
**Female**	25	(32.1)
**Smoking Status**		
**Never-smoker**	39	(50.0)
**Smoker**	39	(50.0)
**Histology**		
**Adenocarcinoma**	33	(42.3)
**Squamous cell carcinoma**	26	(33.3)
**Other**	19	(24.4)
**TNM Stage**^a^		
**I**	64	(82.1)
**II**	5	(6.4)
**III**	9	(11.5)

a TNM stage determined after the surgery.

For the 78 lung cancer specimens from surgical resection, we further classified the HTRA3 expression scores, denoting scores of 0 as negative expression, 1 to 5 as low expression and 6 to 12 as high expression, given that the median semi-quantitative score was 6. Under this classification scheme, 40 (51.3%), 32 (41.0%) and 6 (7.7%) cases were found to have negative, low and high expression scores, respectively. We examined the possible relation between the HTRA3 expression score and various clinical characteristics, but no significant association was found with smoking history, tumor histology, clinical tumor stage, T value or N value. These results are summarized in Supplementary Table 1.

### Correlation of HTRA3 expression with tumor recurrence

**Figure 3:**
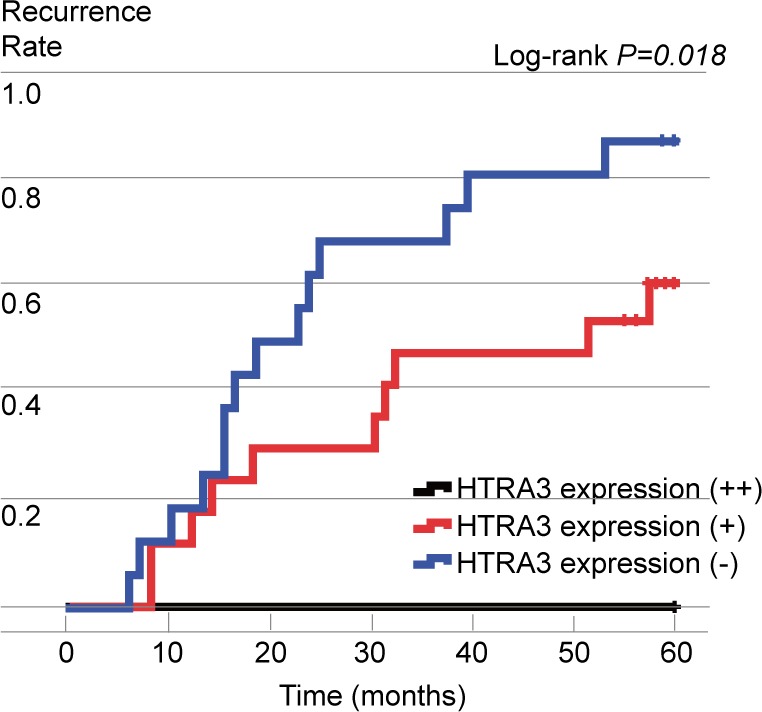
Postoperative recurrence curves of patients in the three groups, stratified according to HTRA3 protein expression

Among the NSCLC patients enrolled, postoperative recurrence was identified in 24 cases (30.8%) during the five-year follow-up period. Of these 24 cases, only one patient developed a loco-regional recurrence, while the other 23 developed a distant metastasis. In detail, tumor relapse was observed in 0 (0%) of the six cases with high expression of HTRA3, 10 (31.2%) of the 32 cases with low expression and 14 (35.0%) of the 40 HTRA3-negative cases. The postoperative recurrence rate was significantly lower in the high-HTRA3 group than in the low and negative groups (*P* = 0.044, 0.029, respectively). The comparison of HTRA3 expression and postoperative recurrence is shown in Table 2. The recurrence curves of the patients in the different groups are depicted in Figure 3.

**Table 2:** The correlation of HTRA3 expression and postoperative recurrence

5-year Follow-up Time	HTRA3 Expression							
	++		+		—		*P*^a^	*P*^b^
	*N*	%	*N*	%	*N*	%		
**Recurrence**	0	0	10	31.2	14	35.0	0.044	0.029
**No Recurrence**	6	100	22	68.8	26	65.0		

a Comparison of postoperative recurrence between groups with high (++) and low (+) expression of HTRA3

b Comparison of postoperative recurrence between groups with high (++) and negative (−) expression of HTRA3

### The influence of HTRA3 expression on disease-free survival and overall survival

**Figure 4:**
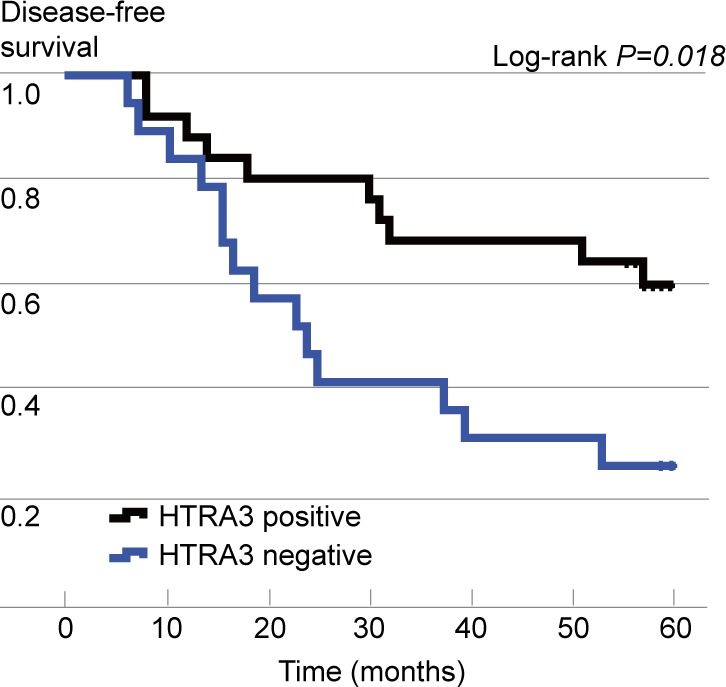
Kaplan-Meier curves of disease-free survival (DFS), stratified according to HTRA3 protein expression

According to the Kaplan-Meier survival analysis, the median DFS was significantly longer in HTRA3-positive patients than in HTRA3-negative patients (*P* = 0.018, Figure 4). Additionally, patients in the high-HTRA3 group had a relatively longer DFS period than those in the low-HTRA3 (*P* = 0.050) and HTRA3-negative group (*P* = 0.009). The DFS was longer in patients with low expression than in those negative for HTRA3, but the results did not reach statistical significance due to the limited sample size (*P* = 0.123). In the multivariate Cox proportional hazards regression model, expression of HTRA3 protein, gender, smoking status, pathology and tumor stage were used as covariates. HTRA3 expression was significantly associated with DFS (hazard ratio [HR] 0.39, 95%CI 0.16-0.95, *P* = 0.038), demonstrating that HTRA3 is an independent prognostic factor of DFS in NSCLC.

Of the 78 patients, 20 (25.6%) died within five years of the operation: 19 died of cancer-related causes and one died of other causes. The five-year overall and disease-specific survival rates were 74.4% and 75.6%, respectively. Kaplan-Meier univariate analysis revealed that OS was longer in HTRA3-positive patients than in HTRA3-negative patients (*P* = 0.025). However, no significant association between HTRA3 expression and OS was observed in the multivariate analysis (shown in Table 3).

**Table 3:** Multivariate analysis of predicting factor for disease-free survival and overall survival

Factors		Disease-free Survival			Overall Survival		
		HR	95%CI	*P*	HR	95%CI	*P*
**Gender**							
	**Male**	0.80	0.24-2.66	0.72	0.39	0.08-1.94	0.25
	**Female**						
**Smoking Status**							
	**Never-smoker**	1.19	0.39-3.65	0.76	0.51	0.13-2.00	0.33
	**Smoker**						
**Histology**							
	**Adenocarcinoma**	1.54	0.57-4.19	0.40	2.19	0.61-7.85	0.23
	**Others**						
**Tumor Stage**							
	**I**	0.66	0.24-1.81	0.42	0.88	0.23-3.30	0.84
	**II or III**						
**HTRA3 Expression**							
	**positive**	0.39	0.16-0.95	0.04	0.39	0.14-1.08	0.07
	**negative**						

### Lung cancer cell invasiveness after HTRA3 overexpression or knockdown

**Figure 5:**
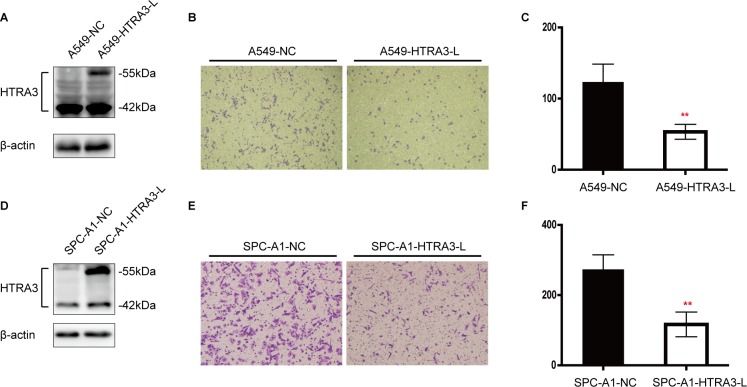
Effect of forced HTRA3 expression on cell invasiveness in NSCLC **A.** and **D.** Overexpression of HTRA3-L in stably transfected A549 and SPC-A1 cell lines was confirmed by Western blotting. **B.** Cell invasion of A549-NC and A549-HTRA3-L cells was examined with the Transwell assay. **E.** Cell invasion of SPC-A1-NC and SPC-A1-HTRA3-L cells was examined with the Transwell assay. **E.** and **F.** Quantification of invasive cells. The bars indicate the mean ± SD. **P < 0.01.

To validate our clinical findings and clarify the specific function of HTRA3 in NSCLC, we investigated whether HTRA3 would suppress or enhance lung cancer cell invasion. Assessment of endogenous HTRA3 expression revealed that the long isoform (HTRA3-L) was expressed at higher levels in normal human small airway epithelial cells (SAECs) than in lung cancer cell lines, whereas the expression of the short isoform (HTRA3-S) did not differ significantly between normal and cancerous cells(Supplementary Figure 2). Thus, we established A549 and SPC-A1 cell lines stably overexpressing *HTRA3-L*. The A549 cells transfected with pLenti-HTRA3-L or empty pLenti were named A549-HTRA3-L andA549-NC, respectively, and the SPC-A1 cells were named in like manner. In Western blot analysis, HTRA3-L expression was significantly higher in A549-HTRA3-L and SPC-A1-HTRA3-L cells than in their respective vector control cells (Figure 5A and 5D).

We next evaluated the invasive capabilities of stably transfected cells using a Matrigel invasion assay. Significantly fewer A549-HTRA3-L cells than A549-NC cells invaded through basement membrane-like Matrigel barriers (Figure 5B and 5C). This finding was also recapitulated in the SPC-A1 cell line, where overexpression of HTRA3-L attenuated tumor cell invasion (Figure 5E and 5F).

In another experiment, two different sequences of siRNA targeting human *HTRA3* were transiently transfected into the A549 cell line. Simultaneous depletion of *HTRA3-L* and *HTRA3-S* enhanced cell invasion in a Transwell assay (Supplementary Figure 3A, 3B and 3C). We also generated siRNA sequences to disrupt *HTRA3-L* specifically, and found that they had the same effect on cell invasion as the combined treatment (Supplementary Figure 3D, 3E and 3F).

## DISCUSSION

Our current data indicated that HTRA3 expression was downregulated in surgically excised cancer tissues compared to normal lung tissues, suggesting that HTRA3 may be an important tumor suppresser in NSCLC. In a previous study, HTRA3 expression was found to decrease with increasing stages of endometrial cancer [13]. In breast cancer, the expression of HTRA3 was significantly lower in stage III than in stages I and II [19]. More recently, HTRA3 levels were found to correlate with the malignant subtypes of ovarian cancers [20]. Although the function of HTRA3 in lung cancer has not been fully identified, a previous study suggested that HTRA3 is a mitochondrial protease that promotes etoposide- and cisplatin-induced cytotoxicity by activating the apoptotic pathway [16]. In addition, the expression of HTRA3 was reduced in smoking-related lung cancers, and this was shown to result from methylation within its first exon [21]. However, none of these reports investigated the relationship between HTRA3 expression and the prognosis of postoperative NSCLC patients. In our study, immunohistochemistry was performed for all the lung cancer tissues from surgical resection. For the first time, we demonstrated that HTRA3 expression negatively correlates with postoperative recurrence and is an independent predictor of DFS in NSCLC.

HTRA3 was first characterized as a pregnancy-related protease that is upregulated during placental development [22,23]. HTRA3 negatively regulates trophoblast cell invasion and migration, raising the possibility that HTRA3 could have a similar effect on cancer cell invasion and metastasis [24,25]. Indeed, in breast cancer, the expression of HTRA3 negatively correlated with lymph node metastasis [19]. On the other hand, a study in oral squamous cell carcinoma indicated that HTRA3 mRNA and protein levels were elevated specifically in invasive tumors compared with premalignant lesions [26]. Further, due to its proteolytic activity, HTRA3 is able to digest some components of the extracellular matrix, which facilitates tumor invasion and metastasis [27,28]. Therefore, in this study, we further investigated whether the expression of HTRA3 in NSCLC cell lines would inhibit or promote tumor cell invasion. Our data showed that ectopic expression of *HTRA3* in A549 and SPC-A1 cells attenuated chemotactic invasion, while depletion of endogenous HTRA3 significantly increased the cell invasion potential. These data suggest the possibility that HTRA3 inhibits tumor invasiveness in NSCLC.

HTRA3 can bind to several members of TGF-β family (BMP4, TGF-β1) and inhibit TGF-β signaling [29,30]. TGF-β signaling is an important stimulator of tumor cell motility and the main inducer of the epithelial - mesenchymal transition (EMT) at the late stages of tumorigenesis [31,32]. In endometrial cancer, a negative correlation between HTRA3 and TGF-β1 protein levels was identified [14]. Thus, it is possible that HTRA3 inhibits TGF-β function in its activation of cancer cells motility. However, further investigation is required to clarify the mechanism whereby HTRA3 may suppress invasion and metastasis in NSCLC.

Both variants of HTRA3 (long and short) have unique structural elements. Recently, Glaza et al.[33] demonstrated that neither the PDZ domain of HTRA3-L nor the seven-residue C-terminal sequence of HTRA3-S was essential for their protease activity. However, the PDZ domain was necessary for the formation of the HTRA3 trimer, and performed an important regulatory function. This corroborated the finding that full-length HTRA3-L was more efficient than PDZ-deleted HTRA3-S in promoting cancer cell apoptosis [16]. Our results also suggested that HTRA3-L restrains tumor cell invasion. Expression of the long isoform was significantly lower in NSCLC cell lines than in a normal lung cell line, while the level of the short form did not change much. Consequently, it may be hypothesized that the two variants of HTRA3 have differential specificities and functions in lung cancer progression. Further research is required to elucidate the specific activity of each isoform in the biological context of tumor cells in NSCLC.

The present study had two major implications for NSCLC management. First, the assessment of HTRA3 expression may effectively improve risk stratification and allow the identification of subgroups requiring more attentive follow-up after surgical resection. Second, since HTRA3 is downregulated and proposed to be a tumor suppressor in NSCLC, stimulating HTRA3 activity may be a potential therapeutic strategy with which to inhibit tumor malignancy. Singh et al. have already generated HTRA3 inhibitory and stimulatory monoclonal antibodies, the foundation for the development of therapeutics specifically targeting HTRA3 [34].

In summary, HTRA3 is downregulated in lung cancer and could be both an attractive predictor of postoperative recurrence and a treatment target for a subset of patients with NSCLC. In particular, the present study strongly indicates that HTRA3 negatively regulates lung cancer cell invasiveness. Thus, the underlying mechanism by which HTRA3 suppresses tumor cell motility clearly warrants further investigation in confirmatory studies and in varied tumor types.

## SUPPLEMENTARY MATERIAL FIGURES AND TABLE


