# Supplementary material for: High temperature requirement A3 (HTRA3) expression predicts postoperative recurrence and survival in patients with non-small-cell lung cancer
Source: Oncotarget. 2016 May 4;7(26):40725–34. doi: 10.18632/oncotarget.9173 (PMC5130039; doi:10.18632/oncotarget.9173)
Supplement: Supplementary file 1 [file oncotarget-07-40725-s001.pdf]

High temperature requirement A3 (HTRA3) expression predicts postoperative recurrence and survival in patients with non-small-cell lung cancer

Supplementary Material

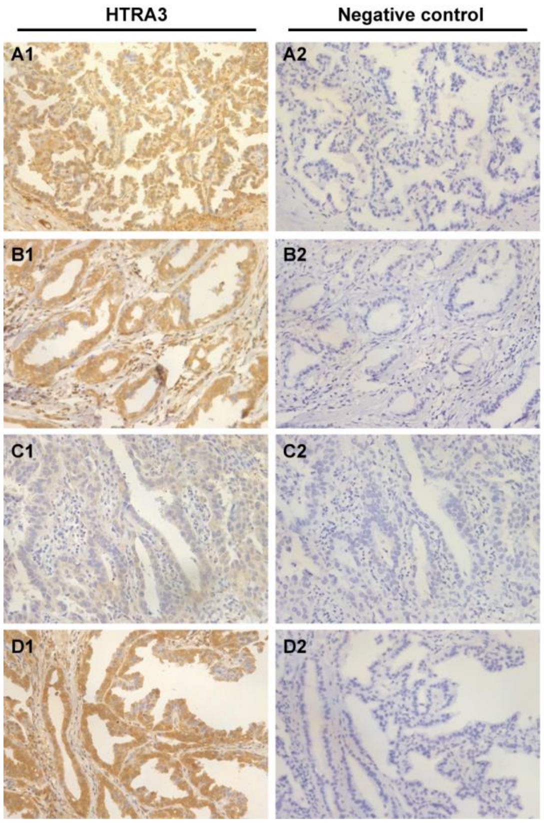

Supplementary Figure1. Immunohistochemical staining of HTRA3 protein in NSCLC specimens and their corresponding negative control

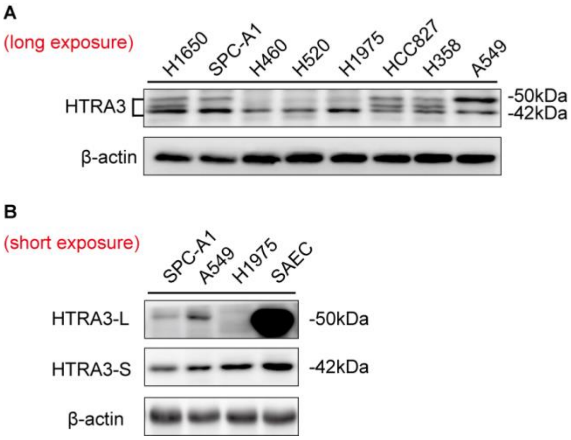

Supplementary Figure2. Expression of HTRA3 protein in normal and lung cancer cell lines, identified by Western blotting

A, HTRA3-L and HTRA3-S protein expression was examined in eight NSCLC cell lines (long exposure). β-actin was used as a loading control. B, Comparison of HTRA3 expression in the normal lung cell line SAEC and lung cancer cell lines (short exposure).

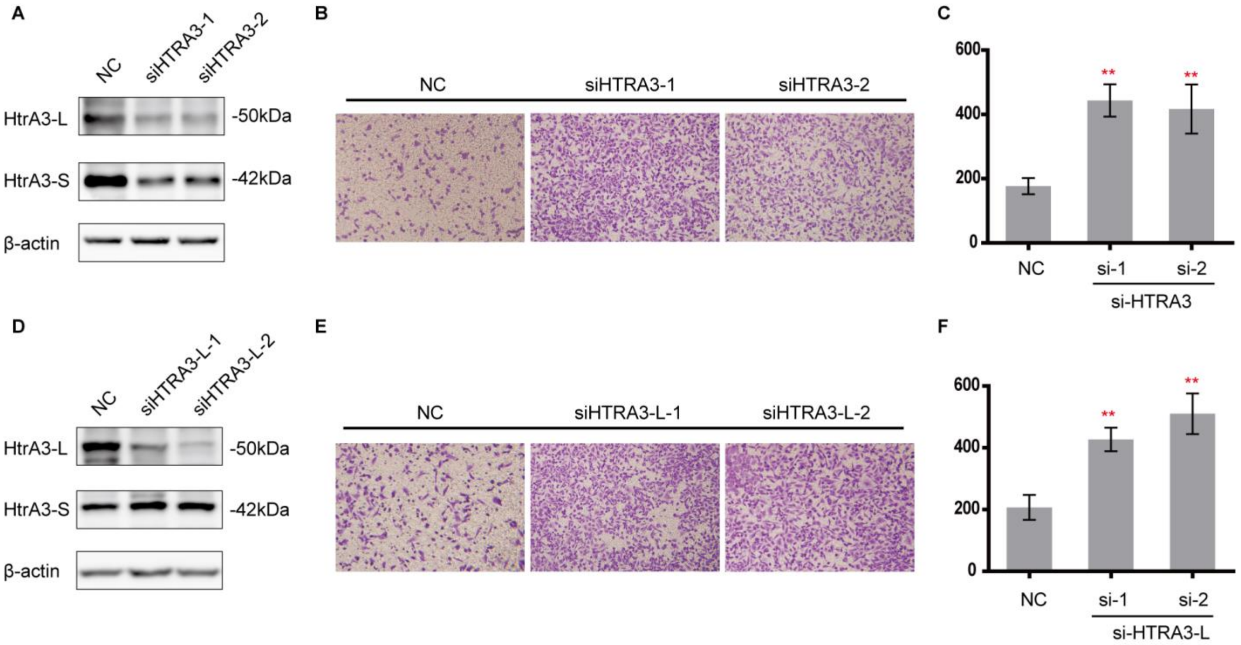

Supplementary Figure3. Effect of depleting HTRA3 expression on cell invasiveness in the A549 cell line
A, Immunoblots show the most effective silencing siRNAs targeting the long and short isoforms of *HTRA3* in A549 cells. B and C, Cell invasion of control and *HTRA3*-RNAi cells was examined with the Transwell assay. Columns, quantification of invasive cells; bars, mean ± SD; ** P<0.01. D, Immunoblots show the silencing siRNAs specifically targeting the long isoform of *HTRA3* in A549 cells. E and F, Cell invasion of control and *HTRA3-L*-RNAi cells was examined with the Transwell assay. Columns, quantification of invasive cells; bars, mean ± SD; ** P<0.01.

Supplement Table1. The Comparison of Patients' Characteristics between Three Groups in IHC Analysis

Characteristics		HtrA3 Expression						*P*
		++		+		—		
		No.	%	No.	%	No.	%	
**Smoking Status**								
	**Never-smoker**	4	66.7	14	43.8	21	52.5	0.527
	**Smoker**	2	33.3	18	56.2	19	47.5	
**Histology**								
	**Adenocarcinoma**	3	50.0	16	50.0	14	35.0	
	**Squamous cell carcinoma**	2	33.3	11	34.4	13	32.5	0.500
	**Other**	1	16.7	5	15.6	13	32.5	
**TNM Stage**								
	**I**	5	83.3	27	84.4	32	80.0	0.887
	**II or III**	1	16.7	5	15.6	8	20.0	
**T value**								
	**T1**	4	66.7	15	46.9	14	35.0	
	**T2**	2	33.3	15	46.9	23	57.5	0.423
	**T3**	0	0	2	6.2	1	2.5	
	**T4**	0	0	0	0	2	5.0	
**N value**								
	**N0**	5	83.3	28	87.5	35	87.5	0.961
	**N1-2**	1	16.7	4	12.5	5	12.5	
